# Effect of Anacardic Acid against Thiram Induced Tibial Dyschondroplasia in Chickens via Regulation of Wnt4 Expression

**DOI:** 10.3390/ani9030082

**Published:** 2019-03-06

**Authors:** Xiong Jiang, Hui Zhang, Khalid Mehmood, Kun Li, Lihong Zhang, Wangyuan Yao, Xiaole Tong, Aoyun Li, Yaping Wang, Jinhuan Jiang, Mujahid Iqbal, Muhammad Waqas, Jiakui Li

**Affiliations:** 1 College of Veterinary Medicine, Huazhong Agricultural University, Wuhan 430070, China; jiangxiongtgc@sina.com (X.J.); dahuilang@webmail.hzau.edu.cn (H.Z.); khalid.mehmood@webmail.hzau.edu.cn (K.M.); likun2008@webmail.hzau.edu.cn (K.L.); zhanglihong@webmail.hzau.edu.cn (L.Z.); yaowangyuan@webmail.hzau.edu.cn (W.Y.); tongxl@webmail.hzau.edu.cn (X.T.); aoyunli@sina.cn (A.L.); 15827205277@163.com (Y.W.); jinhuan1107j@163.com (J.J.); mujahid@cuvas.edu.pk (M.I.); muhammadwaqas@upr.edu.pk (M.W.); 2 College of Animals Husbandry and Veterinary Medicine, Tibet Agricultural and Animal Husbandry University, Linzhi, Tibet 860000, China; 3 University College of Veterinary & Animal Sciences, Islamia University of Bahawalpur, Bahawalpur 63100, Pakistan; 4 Department of Pathology, Cholistan University of Veterinary and Animal Sciences (CUVAS), Bahawalpur 63100, Pakistan

**Keywords:** Wnt4, anacardic acid, growth plate, chickens, tibial dyschondroplasia

## Abstract

**Simple Summary:**

This study evaluated the ameliorating effect of anacardic acid (AA) in tibial dyschondroplasia (TD) chickens. Our results showed that AA can increase the feed conversion ratio, improve the weight, length and width of the tibia. AA administration restored the antioxidant parameters significantly (*p* < 0.05). The gene expression analysis revealed a decrease in wingless-type member 4 (*Wnt4*) expressions in TD chickens as compared to the control group, while AA treatment up-regulated the Wnt4 expression. The present study demonstrates that the AA plays an important role to prevent the lameness and restore the size of the tibial growth plate of chickens by regulating the expression of Wnt4.

**Abstract:**

Tibial dyschondroplasia (TD) is a tibia bone problem in broilers. Anacardic acid (AA) is a traditional Chinese medicine, which is commonly used to treat arthritis in human. The purpose of the present study is to investigate the effect of AA against TD. A total of 300 day-old poultry birds were equally divided and distributed into three different groups: Control, TD and AA groups. The results showed that the feed conversion ratio was significantly lower in the TD group than control chickens. The tibia bone parameters including weight, length and width were of low quality in TD chickens, while the width of the tibial growth plate was enlarged remarkably. Whereas, in the AA treatment group, the tibia bone parameters showed improvement and tend to return to normal. The antioxidant parameters level of glutathione peroxidase (GSH-Px), superoxide dismutase (SOD), total and antioxidant capacity (T-AOC) was significantly decreased, while malondialdehyde (MDA) level was increased significantly in TD affected chickens. AA administration restored the antioxidant parameters significantly. The gene expression revealed a decrease in Wnt4 expression in TD chickens as compared to control chickens, while AA treatment up-regulated the Wnt4 expression. The present study demonstrates that the AA plays an important role to prevent the lameness and restore the size of tibial growth plate of chickens by regulating the expression of *Wnt4*.

## 1. Introduction

Tibial dyschondroplasia is a major poultry leg problem in rapidly growing birds, especially chickens around the world, in which growth plate (GP) cartilage presents excessive amount in metaphyseal of tibia [1,2,3]. This dull white GP is characterized by reduced endochondral ossification, tibial metaphyseal cartilage cell proliferation, a vascular and non-mineralized cartilage wedge, which ultimately leads to overt locomotion problems [4,5]. In 1978, this disease was first described in turkeys and a recent statistical study revealed that almost 30% of bone disease was Tibial dyschondroplasia (TD) in chickens and turkeys with high morbidity, mortality and the condemnation at the processing plant, which is responsible for huge economic losses to the poultry industry [4,5]. Moreover, lameness which is commonly related to TD is also a serious animal welfare issue in broiler chickens. Many studies have been reported that it is caused by a disturbance in vascular development, osteoblast formation and genes related to bone vascularization and mineralization [3,6].

Wingless-type member 4 (Wnt4), helps in cell proliferation, and differentiation in humans and other species [7]. Wnt4 is a secreted protein from osteoblasts and has a significant effect to inhibit bone resorption [8]. Traditional Chinese herbal medicines have been proven to be effective for the treatment of various diseases and conditions in both humans and animals. History goes back to thousands of years of practice with safe and wide sources. Along with other benefits, the mandatory action makes such drugs as a first choice in drug screening [9]. 

Anacardic acid (AA) is a Chinese traditional medicine, which is purified from the bark of *Amphiptery giumadstringens*. Recent research showed that AA could inhibit the proliferation of different kinds of tumor cells (lung, liver and prostate cancer). It is also capable of inducing apoptosis [10]. Moreover, some researchers found that sumac acid has played a good role against bone arthritis in mice through the regulation of matrix metalloproteinases (MMPs) expression for the treatment of osteoarthritis. Wnt4 is an important secreted protein; secreted more from osteoblasts confirmed a protective effect against chronic inflammation and bone loss. Considering the characteristics of AA, we hypothesized that AA could better treat TD via improving Wnt4 expression. Therefore, the main objective of this study is to check the therapeutic effect of AA on TD affected chickens and to explore its molecular mechanisms.

## 2. Materials and Methods

### 2.1. Ethical Approval

All animal experiments were carried out according to the guidelines of the Animal Care, Health and Supervisory Committee of Huazhong Agricultural University, China (approval no. 31273519).

### 2.2. Chickens Husbandry

Three hundred day-old Arbor Acres chickens (weighing 49 ± 7 g) were brought from a market and raised under standard temperature and humidity conditions. All the chickens were fed standard ad libitumdiet according to the guideline of the National Research Council (1994). After 3 days, the chicks were randomly distributed between the control group (n = 100) with a standard diet, and thiram group (n = 200). Thiram group was offered a standard diet with the addition of thiram (50 mg/kg ofdiet). On day 7, one hundred birds from thiram group were separated and named as AA group, which was fed on standard feed along with AA @5 mg/kg/day until the end of the experiment. 

### 2.3. Morphological, Production Parameters Analysis and Sample Collection

All the groups were reared for 18 days, while data regarding the number of lame birds in each group was recorded on daily basis. Twenty chickens from each group were randomly selected and slaughtered on days 7, 10, 14 and 18. After euthanizing, the tibia bones were measured for morphological examination including weight, length, and width of tibial bone and size of tibial GP by an electronic balance and Digital Calipers respectively. The TD score was calculated in all the groups according to Pines et al. [11]. From each group, liver, blood and tibia bones collected and immediately stored at −70 °C for further analysis. 

### 2.4. Hematoxylin and Eosin (H and E) Staining

The tibia bone samples were decalcified in Ethylene diaminetetraacetic acid (EDTA) fluid (10%), dehydrated alcohol solutions, cleaned in xylene and fixed in paraffin wax. The sections were sliced to prepare the slides. In the end, histological sections were stained with H and E to observe the pathological changes under the light microscope according to a previous study [12]. The Image-Pro^®^ Plus 6.0 (Media Cybernetics, Georgia, USA) was used to analyze the blood vessel area.

### 2.5. Measurement of SOD, MDA Contents, GSH-Px, and T-AOC Activity

The analysis of superoxide dismutase (SOD), malondialdehyde (MDA) contents, glutathioneperoxidase (GSH-Px), and total antioxidant capacity (T-AOC) was performed using the commercial assay kit (Nanjing Institute of Biological Engineering Inc. Jiangsu, China) as mentioned in a previous study [13]. Briefly, the liver samples in each group were rinsed, measured and then homogenized for 10 min. After that, centrifugation was performed @ 3000 rpm for 10 min at 4 °C. 

### 2.6. Reverse Transcription Quantitative-Polymerase Chain Reaction (RT-qPCR)

Total RNA of primary tibial growth plates was extracted using the TRIzol (Google biological, Wuhan, China), and then converted into cDNA by using a cDNA kit (Tian Gen, China) as per manufacturer’s recommendations.

The primers for the Wnt4 gene were designed with Primer Premier Software (version 5.0, Premier, Canada) as F, AGCTCTTAGACTGGCTTGTGT; R, TGCTCGGGTCAGTCAAACTC. The RT-qPCR was run in quadruplex by using the specific primers and with cycling protocol of Shahzad et al. [6]. Glyceraldehyde 3-phosphate dehydrogenase (GAPDH) was used as a control and housekeeping gene.

### 2.7. Western Blot Analysis 

The growth plate of individual tissues was homogenized and kept at 4 °C for 2 h and then centrifuged. After that, concentrations of the total proteins were determined according to our previous study Zhang et al. [14].

### 2.8. Statistical Analysis 

The differences and means among various groups were compared through Analysis of variance (NOVA) and student *t*-test to compare by using SPSS 19.0 software (SPSS Version 17.0. Chicago, IL, USA). The data are presented as means ± S.E.M (standard error of means).

## 3. Results

### 3.1. Effects of AA on Lameness, Growth-Plate and Morphology

**Figure 1:**
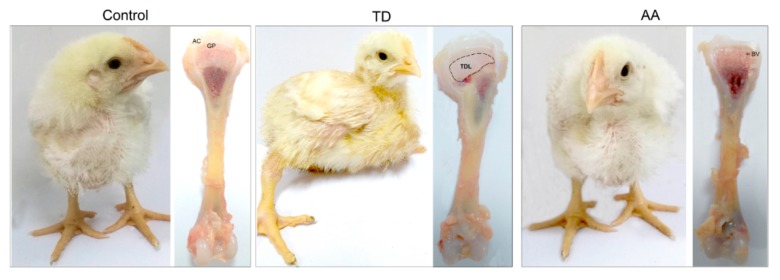
The effects of AA on TD dependent lameness and tibia bone. AC= articular cartilage, BV = blood vessel, TDL = tibial dyschondroplasia lesion, GP = growth plate. AA = Anacardic acid, TD = tibial dyschondroplasia.

The visual examination of chickens showed depression, weakness, lameness, feeding difficulties and poor body condition in TD chickens. While, after AA medicine treatment, the chickens started to walk properly with no lameness. The size of the tibial growth plate (GP) was markedly increased in the thiram group compared with the control group. The AA medicine administration significantly reduced the size of GP compared with the TD group (Figure 1).

### 3.2. TD Score

**Figure 2:**
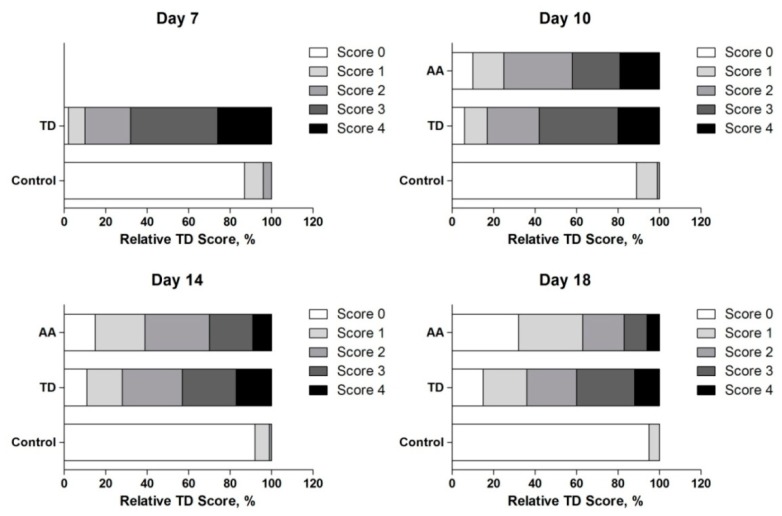
Effect of thiram and AA on TD scores in chickens. TD severity score on day 7, 10, 14 and 18. Compared with the TD group, the AA gave significant decreased in TD, especially on day 14 and 18. AA = Anacardic acid, TD = tibial dyschondroplasia.

The results showed that thiram can induce TD; almost 90% of chickens were found lame after day 4 of thiram administration. In the control group, tibia had almost normal phenotype and less average TD score. However, AA group showed that chicken restored the TD pathogenesis after the administration of AA and more than 30% of chickens were found healthy and more than 60% of chickens showed less TD (score 1) on day 18 (Figure 2).

### 3.3. Tibial Parameters Evaluation 

**Figure 3:**
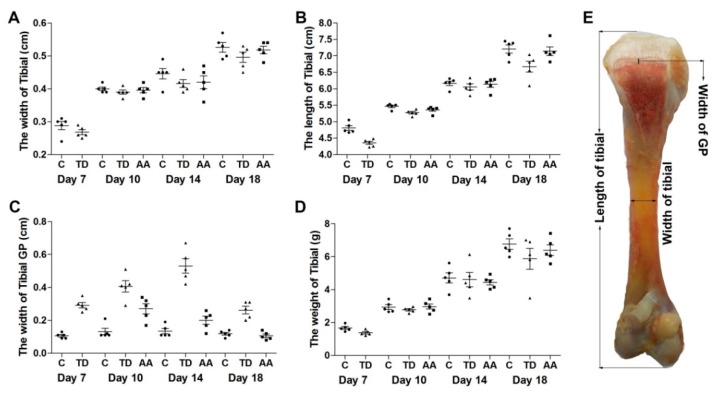
Effects of AA on thiram-induced tibial dyschondroplasia chickens via overall tibial parameters analysis. (**A**–**D**) correlation analysis among the length, width, weight of tibia and the size of the tibial growth plate were analyzed using the Pearson test. C = control; AA = Anacardic acid, TD = tibial dyschondroplasia.

The tibial parameters like tibial length, width, weight and width of GP in chickens were recorded among all the groups on days 7, 10, 14 and 18. An overall performance parameters analysis of tibia bone showed that there was a positive trend among length of tibia, width and weight of tibia from day 7 to day 18, but the difference was not significant. Meanwhile, the width of GP was significantly enlarged (*p* < 0.05) in the TD group than the control group. However, with the administration of AA, GP width was decreased significantly in the AA group compared with the TD group (Figure 3). 

### 3.4. Histological Examination of Tibial Growth Plate 

**Figure 4:**
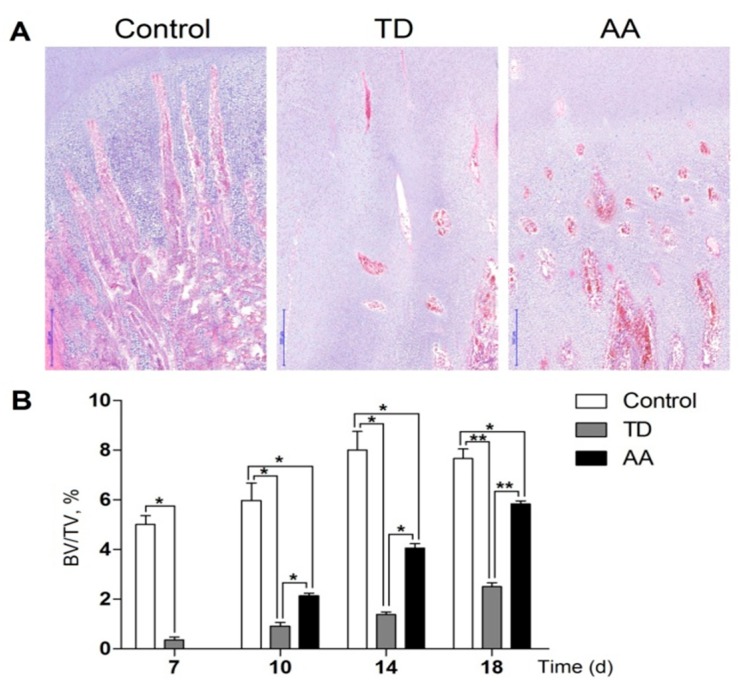
The H and E analysis of normal GP indicates regular columns and cells surrounded by many blood vessels. (**A**) H and E analysis. (**B**) Trabecular bone volume assay of different groups. Growth plates in the TD group were less vascularized and AA restored angiogenesis. * *p* < 0.05; ** *p* < 0.01. AA = Anacardic acid, TD = tibial dyschondroplasia.

Tibial growth plates were evaluated with hematoxylin and eosin (H and E) and there was a prominent difference between TD and control group. The columns were well-conserved and surrounded with a large number of blood vessels in the hypertrophic and proliferative zone of GP in the control group. In TD affected birds, the histological examination showed that the GP revealed necrosis and few blood vessels with immature cartilage cells and cells were arranged tightly. Whereas administration of AA medicine resulted in new blood vessels formation, the width of the hypertrophic region reversed and angiogenesis was observed significantly (Figure 4A). The blood vision analysis indicated that there was a significant difference between TD and AA group during the entire study period on various days (Figure 4B).

### 3.5. Liver Antioxidant Levels 

**Figure 5:**
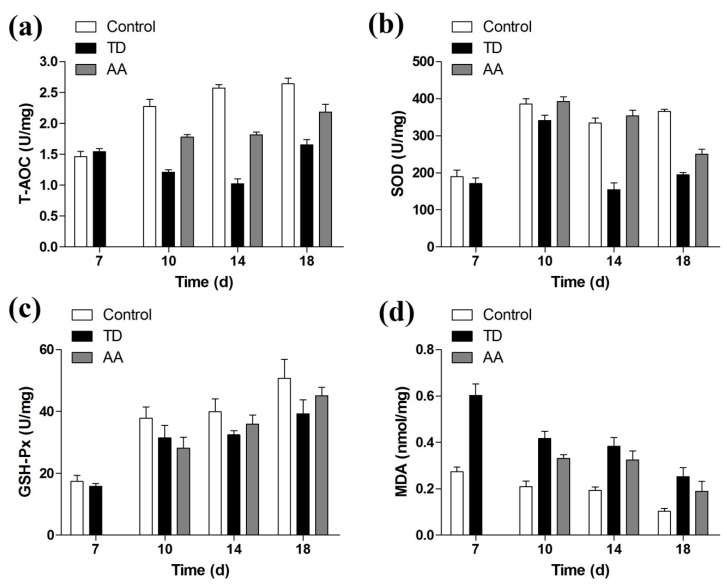
Effect of AA on liver antioxidant activities in broilers at 7, 10, 14 and 18d.The data are shown as the mean ± SEM. (**a**) T-AOC. (**b**) SOD. (**c**) GSH-Px (**d**) MDA. AA = Anacardic acid, TD = tibial dyschondroplasia.

Our results indicated that compared to the control group, the level of SOD, T-AOC and GSH-Px were significantly less (*p* < 0.05) in the TD group. Whereas, the level of MDA was enhanced in the TD group as compared to control group chickens. However, the AA treatment reversed these variations, the SOD, T-AOC and GSH-Px level was increased and MDA level was decreased significantly (*p* < 0.05) as shown in Figure 5. 

### 3.6. Effects of AA on Expression of Wnt4 Gene in Growth Plate

**Figure 6:**
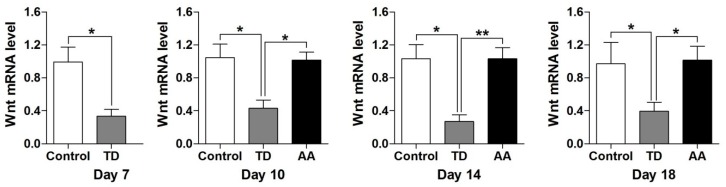
RT-qPCR analysis indicates expression of Wnt4 gene in control, thiram and AA groups on various days. Data are presented as the means ± SEM, * *p* < 0.05; ** *p* < 0.01. AA = Anacardic acid, TD = tibial dyschondroplasia.

**Figure 7:**
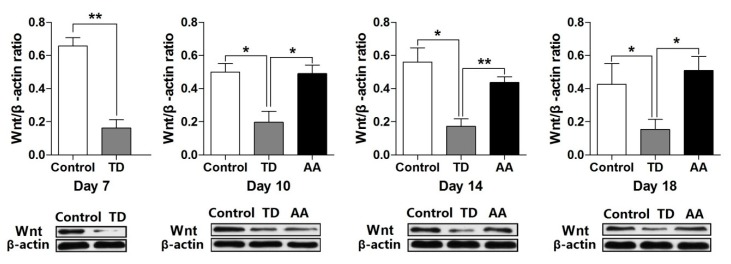
Wnt4 protein expression levels were analyzed in tibial growth plate on various days in Control, Thiram and AA groups. Protein levels of Wnt4 were detected by Western blot analysis. Data are presented as the means ± SEM; * *p* < 0.05; ** *p* < 0.01. AA = Anacardic acid, TD = tibial dyschondroplasia.

RT-qPCR and western blot were employed to investigate the expressions of Wnt4 mRNA levels in the growth plate. The overall expression levels of Wnt4 were decreased significantly in TD affected chickens as compared to the control group. While the mRNA level of that gene was increased on day 10, 14 and 18 after the administration of AA (Figure 6). Furthermore, the western blot analysis results were also parallel to gene expression results (Figure 7).

## 4. Discussion

Tibial dyschondroplasia is mainly atibiotarsal bone abnormality, which is common in fast-growing birds especially in chicken and turkey worldwide [15]. It is characterized by endochondral ossification, a mass of avascular opaque white cartilage wedge in metaphysical part of tibiotarsus and tarsometatarsus bones [16,17]. The mechanism being responsible for the development and the treatment of these lesions is still unclear [5]. We checked the pathological changes in the tibial growth plate by histological assessments. According to our results, GP has a circular arc of cartilage with uniform thickness and smooth edges in normal broilers; while the growth plate cartilage showed non-vascular, non-mineralized white cartilaginous mass in TD affected chickens. This makes it very easy for visual examination. This cartilaginous mass ultimately results in movement problem and obstacles in standing. 

Wnts are important for the mesenchymal precursor cells [18,19]. Adipogenesis disturbs the normal process of osteogenesis [20]. Wnt4 helps in cell proliferation, and differentiation in human and other species [7], it protects the bone from resorption and stops the osteoclast formation [21,22]. Wnt4 was also reported to associate with femoral neck BMD and lumbar spine in previous studies [23,24]. The present study also investigates causality between common and rare genetic variations inWnt4 in TD affected chickens. In our study, we found that the Wnt4 level was decreased significantly in TD chickens as compared to the controlgroup on day 7 to day 14. However, the gene expression levels were restored by AA on day 18 compared with TD chickens. Our results indicated that AA has a protective effect over bone loss and bone resorption.

Thiram is widely used in pesticide practices in agriculture region, but it is also used to induce TD [14,23]. Some reports showed that it interacts with GP and disturb the metabolism and the development of chondrocytes to cause TD [25,26,27]. On the other hand, it is reported that thiram has noxious effects on liver [4,28,29,30], which release MDA as an end product during lipid peroxidation and ultimately result in oxidative imbalance [31,32]. Li et al. [33] reported that thiram destroyed the oxidative balance through decreasing SOD and GSH-Px and induced oxidative processes in chicken liver such as lipid peroxidation. Altogether, higher MDA concentrations affected the liver function and caused TD in broilers. In this study, the level of SOD, T-AOC and GSH-Px were significantly less (*p* < 0.05) in the TD group compared to the control group. Whereas, the level of MDA was increased in TD chickens as compared to normal chickens. On the other hand, with the use of AA, the level of antioxidants parameter was significantly increased, while oxidative stress was decreased significantly (*p* < 0.05).

## 5. Conclusions

The present study demonstrates that AA plays an important role in preventing the lameness and restoring the size of tibial growth plate of chickens by regulating the expression of Wnt4. 

## References

[1] Mehmood K., Zhang H., Iqbal M.K., Rehman M.U., Shahzad M., Li K., Huang S., Nabi F., Zhang L., Li J. (2017). In Vitro effect of apigenin and danshen in tibial dyschondroplasia through inhibition of heat-shock protein 90 and vascular endothelial growth factor expressions in avian growth plate cells. Avian Dis..

[2] Mehmood K., Zhang H., Li K., Lei W., Rehman M.U., Nabi F., Iqbal M.K., Luo H., Shahzad M., Li J. (2018). Effect of tetramethylpyrazine on tibial dyschondroplasia incidence, tibial angiogenesis, performance and characteristics via HIF-1α/VEGF signaling pathway in chickens. Sci. Rep..

[3] Nabi F., Li K., Shahzad M., Han Z., Zhang D., Liu J., Li K. (2016). Gambogic acid inhibits hsp90 expressions in thiram-induced tibial dyschondroplasia. Pak. Vet. J..

[4] Dan H., Simsa-Maziel S., Hisdai A., Sela-Donenfeld D., MonsonegoOrnan E. (2009). Expression of matrix metalloproteinases during impairment and recovery of the avian growth plate. J. Anim. Sci..

[5] Leach R., Monsonego-Ornan E. (2007). Tibial dyschondroplasia 40 years later. Poult. Sci..

[6] Shahzad M., Liu J., Gao J., Wang Z., Zhang D., Nabi F., Li J.K. (2015). Differential expression of extracellular matrix metalloproteinase inducer (EMMPRIN/CD147) in avian tibial dyschondroplasia. Avian Pathol..

[7] Timmermans-Sprang E.P.M., Gracanin A., Mol J.A. (2017). Molecular Signaling of Progesterone, Growth Hormone, Wnt, and HER in Mammary Glands of Dogs, Rodents, and Humans, New Treatment Target Identification. Front. Vet. Sci..

[8] Algandaby M.M., Breikaa R.M., Eid B.G., Neamatallah T.A., Abdel-Naim A.B., Ashour O.M. (2017). Icariin Protects against Thioacetamide-induced Liver Fibrosis in Rats, Implication of Anti-angiogenic and Anti-autophagic Properties. Pharmacol. Rep..

[9] Nambiar J., Bose C., Venugopal M., Banerji A., Patel T.B., Kumar G.B., Nair B.G. (2016). AA inhibits gelatinases through the regulation of Spry2, MMP-14, EMMPRIN and RECK. Exp. Cell Res..

[10] Seong Y.A., Shin P.G., Yoon J.S., Yadunandam A.K., Kim G.D. (2014). Induction of the endoplasmic reticulum stress and autophagy in human lung carcinoma A549 cells by AA. Cell Biochem. Biophys..

[11] Pines M., Hasdai A., Monsonego-Ornan E. (2005). Tibial dyschondroplasia–tools, new insights and future prospects. World’s Poult. Sci. J..

[12] Mehmood K., Zhang H., Iqbal M.K., Rehman M.U., Li K., Huang S., Shahzad M., Nabi F., Mujahid I., Li J. (2018). Tetramethylpyrazine mitigates toxicity and liver oxidative stress in tibial dyschondroplasia chickens. Pak. Vet. J..

[13] Zhang H., Chang Z., Mehmood K., Rao Z.A., Nabi F., Rehman M.U., Wu X., Tian X., Yuan X., Li Z. (2018). Nano copper induces apoptosis in pk-15 cells via a mitochondria-mediated pathway. Biol. Trace Elem. Res..

[14] Zhang H., Khalid M., Jiang X., Yao W.Y., Mujahid I., Li K. (2018). Effect of icariin on tibial dyschondroplasia incidence, tibial characteristics by regulating P2RX7 in chickens. Biomed. Res. Int..

[15] Simsa S., Hasdai A., Dan H., Ornan E.M. (2007). Induction of tibial dyschondroplasia in turkeys by tetramethylthiuram disulfide (thiram). Poult. Sci..

[16] Shim M.Y., Karnuah A.B., Anthony N.B., Pesti G.M., Aggrey S.E. (2012). The effects of broiler chicken growth rate on valgus, varus, and tibial dyschondroplasia. Poult. Sci..

[17] Tian W.X., Zhang W.P., Li J.K., Bi D.R., Guo D.Z., Pan S.Y., Zhang Y.H., Qin P. (2009). Identification of differentially expressed genes in the growth plate of broiler chickens with thiram-induced tibial dyschondroplasia. Avian Pathol..

[18] Aziz A., Miyake T., Engleka K.A., Epstein J.A., Mcdermott J.C. (2009). Menin expression modulates mesenchymal cell commitment to the myogenic and osteogenic lineages. Dev. Biol..

[19] Zhu X., Zhu H., Zhang L., Huang S., Cao J., Ma G., Feng G., He L., Yang Y., Guo X. (2012). Wls-mediated wnts differentially regulate distal limb patterning and tissue morphogenesis. Dev. Biol..

[20] Millet L. (2001). Role of wnt pathway in the determination of human mesenchymal stem cells into preadipocytes. J. Mol. Endocrinol..

[21] Yu B., Chang J., Liu Y., Li J., Kevork K., Al-Hezaimi K., Graves D.T., Park N.H., Wang C.Y. (2014). Wnt4 signaling prevents skeletal aging and inflammation by inhibiting nuclear factor-kappa B. Nat. Med..

[22] Zhang H., Mehmood K., Li K., Rehman M.U., Jiang X., Huang S., Wang L., Zhang L., Tong X., Nabi F. (2018). Icariin Ameliorate Thiram-Induced Tibial Dyschondroplasia via Regulation of WNT4 and VEGF Expression in Broiler Chickens. Front. Pharmacol..

[23] Rasaputra K.S., Liyanage R., Lay J.O., McCarthy F.M., Rath N.C. (2010). Tibial dyschondroplasia-associated proteomic changes in chicken growth plate cartilage. Avian Dis..

[24] Zheng Y., Wang C., Zhang H., Shao C., Gao L.H., Li S.S., Yu W.J., He J.W., Fu W.Z., Hu Y.Q. (2016). Polymorphisms in wnt signaling pathway genes are associated with peak bone mineral density, lean mass, and fat mass in chinese male nuclear families. Osteoporos. Int..

[25] Rath N.C., Huff W.E., Huff G.R. (2007). Thiram-induced changes in the expression of genes relating to vascularization and tibial dyschondroplasia. Poult. Sci..

[26] Mehmood K., Zhang H., Jiang X., Yao W., Tong X., Iqbal M.K., Rehman M.U., Iqbal M., Waqas M., Qamar H. (2019). Ligustrazine recovers thiram-induced tibial dyschondroplasia in chickens, involvement of new molecules modulating integrin beta 3. Ecotoxicol. Environ. Saf..

[27] Iqbal M., Hui Z., Khalid M., Aoyun L., Xiong J., Yaping W., Zhang J., Iqbal M.K., Rehman M.U., Yao W. (2018). Icariin: A potential compound for the recovery of tibial dyschondroplasia affected chicken via up-regulating bmp-2 expression. Biol. Proced. Online.

[28] Li J., Bi D.R. (2008). Effects of high dietary vitamin A supplementation on tibial dyschondroplasia, skin pigmentation and growth performance in avian broilers. Res. Vet. Sci..

[29] Zhang H., Khalid M., Jiang X., Yao W.Y., Mujahid I. (2018). Effect of Tetramethyl thiuram disulfide (thiram) in relation to tibial dyschondroplasia in chickens. Environ. Sci. Pollut. Res..

[30] Iqbal M.K., Nabi F., Mehmood K., Rehman M.U., Huang S., Zhang H., Zhang L., Ahmad H.I., Iqbal M., Li J. (2018). Healing of growth plate cartilage by hypoxia Inducible Factor-1α inhibitor apigenin on thiram induced tibial dyschondroplasia. Pak. Vet. J..

[31] Marikovsky M. (2002). Thiram inhibits angiogenesis and slows the development of experimental tumours in mice. Br. J. Cancer.

[32] Perry J.J.P., Shin D.S., Getzoff E.D., Tainer J.A. (2010). The structural biochemistry of the superoxide dismutases. Biochim. Biophys. Acta.

[33] Li J., Bi D., Pan S., Zhang Y. (2007). Effect of diet with thiram on liver antioxidant capacity and tibial dyschondroplasia in broilers. Br. Poult. Sci..

